# QT Prolongation-induced Seizures Masquerading as Depression with Mixed Features

**DOI:** 10.1192/j.eurpsy.2022.1209

**Published:** 2022-09-01

**Authors:** A. Capilla Crespillo, N. Salvat Pujol, D. Palao Vidal, J. Pinzón-Espinosa

**Affiliations:** 1 Consorci Corporació Sanitària Parc Taulí, Psychiatry, Sabadell, Spain; 2 Centro de Investigaci ´on Biom ´edica en Red de Salud Mental (CIBERSAM), Salud Mental, Madrid, Spain; 3 School of Medicine, Universitat Autònoma de Barcelona, Medicine, Cerdanyola del Vallés, Spain; 4 School of Medicina, Clinical Psychiatry, Panamá, Panama; 5 School of Medicine, University of Barcelona, Medicine, Barcelona, Spain

**Keywords:** Emergency psychiatry, bipolar disorders, psychosomatics

## Abstract

**Introduction:**

Severe mental disorders experience premature mortality mostly from physical causes. When a patient with a history of bipolar disorder is admitted to the emergency room (ER) for psychiatric symptoms, these are routinely interpreted as a psychiatric disturbance. However, a careful history should be performed to correctly interpret key clinical information to rule out somatic etiology and establish adequate diagnosis.

**Objectives:**

To describe a patient whose presenting symptoms were misdiagnosed as psychiatric relapse, rather than serious somatic comorbidity debut.

**Methods:**

A 70-year-old man, with a history of type I bipolar disorder and multiple cardiovascular conditions, was admitted to the ER for self-referred nervousness, depressed mood, insomnia, and suicidal thoughts. Symptoms had greatly worsened the previous week to his consultation with paroxysmal episodes of severe anxiety, feelings of strangeness, and sensations of unpleasant odors.

**Results:**

During observation, the patient was found lying down with loss of consciousness, urinary incontinence, and amnesia of the event. Generalized tonic-clonic seizures were observed by neurologists while mental status examination was being performed. After symptoms were oriented as having a neurological etiology, the patient suffered cardiac arrest and defibrillation was required. After admittance to the intensive care unit and inpatient cardiology care, the patient was discharged from the hospital with the diagnosis of ventricular fibrillation due to drug-induced QT prolongation. There was no evidence of mixed depression or seizures once the cardiac dysfunction was identified and treated.

**Conclusions:**

The psychiatric symptoms were the clinical manifestation of a generalized seizure-like activities that were attributed to transient cerebral hypoperfusion secondary to ventricular fibrillation.

**Disclosure:**

No significant relationships.

